# Clinical, Molecular and Functional Investigation on an Infant with Neonatal Intrahepatic Cholestasis Caused by Citrin Deficiency (NICCD)

**DOI:** 10.1371/journal.pone.0089267

**Published:** 2014-02-21

**Authors:** Zhan-Hui Zhang, Wei-Xia Lin, Mei Deng, Shu-Tao Zhao, Han-Shi Zeng, Feng-Ping Chen, Yuan-Zong Song

**Affiliations:** 1 Department of Pediatrics, the First Affiliated Hospital, Jinan University, Guangzhou, Guangdong, China; 2 Central Laboratory, the First Affiliated Hospital, Jinan University, Guangzhou, Guangdong, China; 3 Department of Laboratory Science, the First Affiliated Hospital, Jinan University, Guangzhou, Guangdong, China; Innsbruck Medical University, Austria

## Abstract

**Background and Objective:**

*SLC25A13* analysis has provided reliable evidences for the definitive diagnosis of citrin deficiency (CD) in the past decade. Meanwhile, these studies generated some issues yet to be resolved, including the pathogenicity of *SLC25A13* missense mutations and the mRNA product from the mutation c.615+5G>A. This study aims to investigate the effect of a novel missense mutation on the aspartate/glutamate carrier (AGC) function of citrin protein, and to explore the aberrant transcript from c.615+5G>A in the same CD infant.

**Methods and Results:**

By means of screening for prevalent *SLC25A13* mutations and exons sequencing, the patient proved a compound heterozygote of c.615+5G>A and a novel c.1064G>A (p.Arg355Gln) mutation. An aberrant transcript with retention of the entire intron 6, *r*.[615+1_615+1789ins; 615+5 g>a] (GenBank accession number KJ128074), which was resulted from c.615+5G>A, was detected by RT-PCR and cDNA sequencing. After bioinformatic analyses of the novel missense mutation c.1064G>A, the growth abilities of three *agc1*Δ yeast strains were tested, which had been transformed with recombinant or empty vectors, respectively. Besides the bioinformatically pathogenic evidences, the growth ability of the *agc1*Δ strains transformed with mutant recombinant was the same as with empty vector, but significantly lower than that with normal control in functional analysis.

**Conclusions:**

A CD infant was definitely diagnosed in this paper by a genetic, transcriptional and functional analysis of *SLC25A13* gene. This study provided direct laboratory evidences supporting the splice-site nature of the c.615+5G>A mutation, and the novel c.1064G>A variation, which proved a pathogenic mutation bioinformatically and functionally, enriched the *SLC25A13* mutation spectrum.

## Introduction

Human citrin deficiency (CD) is an autosomal recessive disease entity caused by *SLC25A13* gene mutations [1]. As the liver-type aspartate-glutamate carrier isoform 2 (AGC2), citrin plays roles in the metabolic pathways of aerobic glycolysis, gluconeogenesis, urea cycle, and synthesis of proteins and nucleotides [2]–[6], and the deficiency of citrin leads to a variety of biochemical, metabolomics, medical imaging, hepatohistological and even behavioral alterations [7]–[11]. Up to now, at least three age-dependant clinical phenotypes have been described for this disease entity, i.e. Neonatal Intrahepatic Cholestasis caused by Citrin Deficiency (NICCD, OMIM#605814) in infants, adult-onset citrullinemia type II (CTLN2, OMIM#603471) in adolescents/adults, and Failure to Thrive and Dyslipidemia caused by Citrin Deficiency (FTTDCD), a novel CD phenotype between NICCD and CTLN2 stage which was proposed very recently [12]–[15].

The analyses of *SLC25A13* gene and its transcriptional and/or translational products have provided reliable evidences for the definitive diagnoses of CD patients worldwide [16]–[20]. However, molecular diagnosis of CD patients in the past decade engendered some issues to be resolved. For example, the abnormal mRNA molecule from the mutation c.615+5G>A was not detected yet, making it obscure how this mutation causes formation of inactive citrin protein [17], [21]. Furthermore, most of the reported *SLC25A13* mutations are missense mutations [13], [22], but their pathogenicity was just predicted on the basis of clinical and bioinformatics analysis, and functional study on *SLC25A13* missense mutations still remains rather limited [23], [24].

Very recently, we encountered a new NICCD child in our clinical practice, and *SLC25A13* analysis uncovered a novel missense mutation along with the possible splice-site mutation c.615+5G>A. In this paper, by using an *agc1*Δ yeast model, we investigated the functional effect of the novel mutation, and the aberrant transcript in peripheral blood lymphocytes (PBLs) due to the mutation c.615+5G>A was also explored. We herein reported the clinical, molecular and functional findings.

## Subjects and Methods

### Subjects and Ethics Statement

The research subjects in this study were a male patient (C0165) suspected to have NICCD and his parents as well. The clinical information was collected and described as a case report. This research was carried out with written informed consent from the parents, and has been approved by the Committee for Medical Ethics, the First Affiliated Hospital, Jinan University in China, adhering to the World Medical Association Declaration of Helsinki (WMADH 2008), which was adopted by the 59th WMA General Assembly, Seoul, in October 2008.

### *SLC25A13* Mutation Analysis

Genomic DNA was extracted from peripheral venous blood samples collected from the subjects. Four High-frequency mutations, i.e. c.851_854delGTAT, c.1638_1660dup, c.615+5G>A and IVS16ins3kb, were screened by PCR/LA-PCR and PCR-RFLP. To explore the possible novel mutation, all the 18 exons and their flanking sequences in *SLC25A13* gene were amplified by PCR/LA-PCR, and the products were then sequenced, as described previously [8], [12], [16], [25].

### Bioinformatic Analyses

The conservative property of the amino acid affected by the novel missense mutation was surveyed by using the software ClustalX 2.0 (http://www.ebi.ac.uk/Tools/msa/clustalo/). The amino acid sequence of human citrin was comparatively aligned with those of the homologous proteins in various species including *H. sapiens* (NP_055066.1), *B. Taurus* (DAA30813.1), *X. tropicalis* (AAI59168.1), *A.aegypti* (XM_001651912.1), *S. cerevisiae* (NP_015346.1) and *C.elegans* (NM_064873.3), which were obtained from the NCBI database (www.ncbi.nlm.nih.gov).

To evaluate the structural effect on citrin protein caused by the amino acid change, a three-dimensional (3D) model of the transmembrane segment of citrin protein was constructed by homology modeling with the assistance of the online tool SWISS-MODEL [26], [27] with the bovine mitochondrial ADP/ATP carrier monomers (2c3eA) [28] as a template. Structures were viewed using the software SWISS-Pdb Viewer 4.10.

The software PolyPhen-2 (Polymorphism Phenotyping version 2.2.2, available at http://genetics.bwh.harvard.edu/pph2/) was applied to predict the possible impact of the missense mutation on the structure and function of human protein [29]. If the probabilistic score of one mutation is above 0.85, it will be classified as “probably damaging”, while as “possibly damaging” with the score above 0.15 [29]. Moreover, mutationTaster (http://mutationtaster.org/MutationTaster/index.html) was also used to evaluate the disease-causing potential of the missense mutation. The probability value close to 1 indicates a high ‘security’ of the prediction [30].

### Reverse Transcription-PCR (RT-PCR)

The RT-PCR process was conducted as in our previous publications [13], [16], [25]. Briefly, two milliliters of EDTA anticoagulant peripheral blood was centrifuged over Lymphocyte Separation Medium (LSM, MP) immediately after sampling from the patient and his parents. Peripheral blood lymphocytes (PBLs) were collected according to the manufacturer’s instructions and then homogenized immediately in Trizol (Life Technologies) to extract total RNA following the manufacture’s protocol. cDNAs were synthesized from 2 µg of total RNA in the presence of primer oligo-(dT)_18_. Using the cDNA above as template, PCR was performed with the primer set Ex5F and Ex8R (Table 1), and the temperature profile was 94°C for 3 min followed by 30 cycles of 98°C for 10 s, 60°C for 5 s, 72°C for 2.5 min and a final extension step at 72°C for 10 min. The RT-PCR products were then sequenced to explore aberrant transcript with c.615+5G>A.

**Table 1:** Primer sequences in this study.

Primers	Sequences from 5′ to 3′ end	Purpose
Ex5F	TTTGGACAGACCACAATTCATC	Amplification the transcript from the allelewith c.615+5G>A
Ex8R	CCCCTTGGCTCATATAAATCTG	
LS	**GTGGATAGCTCACTCGATGTAGTTAGCTTTCAGAATGACGTTTGG** TCCCGGAGACGGTCACAGC	Amplification the disruptioncassette
LA	**CTTCTGTTTTCCTTTGAATTTGCGACGATAGAAAATAGTAGGTTG** CCTACAGCGTGAGCATTGAG	
AGC1A	AGTATGCAGCTTTGCTACCG	Detection of the *agc1* gene
AGC1B	CTTGTGAGTCCATCTGTGCC	
JMUS	AGTCGCAGGATTCTCCAGTTCGC	Evaluation of the disruption effect of*agc1* gene
JMDA	AACCCGCAATAGAACCCAAGGAG	
PSLCF	ACCG*GAATTC*ATGGCGGCCGCCAAGGTG	Construction recombinant plasmids
PSLCR	ACCCG*AAGCTT*AGGAGGGATGTTCTTTACTG	
V01F	CTTGTAAAAACTC***A***AATGCAGAACC	Site-directed mutagenesis for p.R355Q
V01R	GGTTCTGCATT***T***GAGTTTTTACAAG	

The nucleotides in italic in the primers PSLCF and PSLCR were introduced restriction sites, those in italic bold in V01F and V01R are introduced point mutations, and those in underlined bold in LS and LA are homologous sequences on both sides of the deletion region in *agc1* gene.

### Yeast Strains and *agc1* Gene Disruption

**Figure 1:**
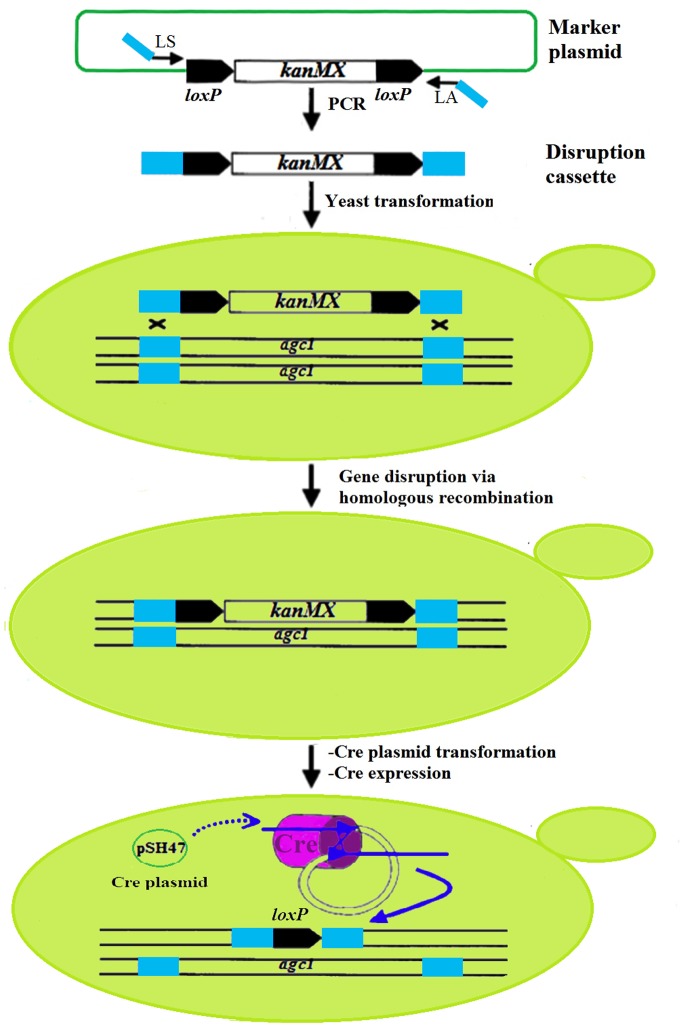
Disruption procedure of the *agc1* gene in yeast strain *Saccharomyces cerevisiae* BY4741. The disruption cassette was produced by PCR amplification using the primer pair LS and LA. The cassette consisted of a selection marker kanMX flanked by two *loxP* sequences adjacent to the 45 bp homologous sequences (dark green box). The *agc1* gene to be deleted was flanked by the same homologous sequences in the yeast strain. After transformation of the disruption cassette and homologous recombination in the yeast cells, selected strains were checked by PCR for correct integration of the cassette and concurrent deletion of *agc1* gene. Then the diploid yeast strain with the genotype *agc1*/*agc1*::loxP-kanMX-loxP was transformed with plasmid pSH47 to express the Cre recombinase, which removed the marker gene and resulted in the genotype *agc1*/*agc1*:: loxP. The same procedure was performed once again, and another allele of *agc1* could also be disrupted, resulting in formation of the genotype *agc1::loxP*/*agc1*::*loxP*.

The yeast strain *Saccharomyces cerevisiae* BY4741 (*MATa; his3*Δ*1; leu2*Δ*0; met15*Δ*0; ura3*Δ*0*) was used to construct a eukaryotic model by disruption of *agc1*, the gene highly homologous to *SLC25A13* in human, by means of the Cre-loxP recombination system with plasmid set of pUG6 and pSH47 (as illustrated in Figure 1). All the primers in this process were listed in Table 1 and all culture media prepared as described previously [31], [32]. The improved LiAc/SS-DNA/PEG procedure was used to transform the disruption cassette or plasmid to yeast cells [33]. The diploid *agc1*-disrupted strain with genotype *agc1::loxP*/*agc1*::*loxP* was designated as BY*agc1*Δ.

**Figure 2:**
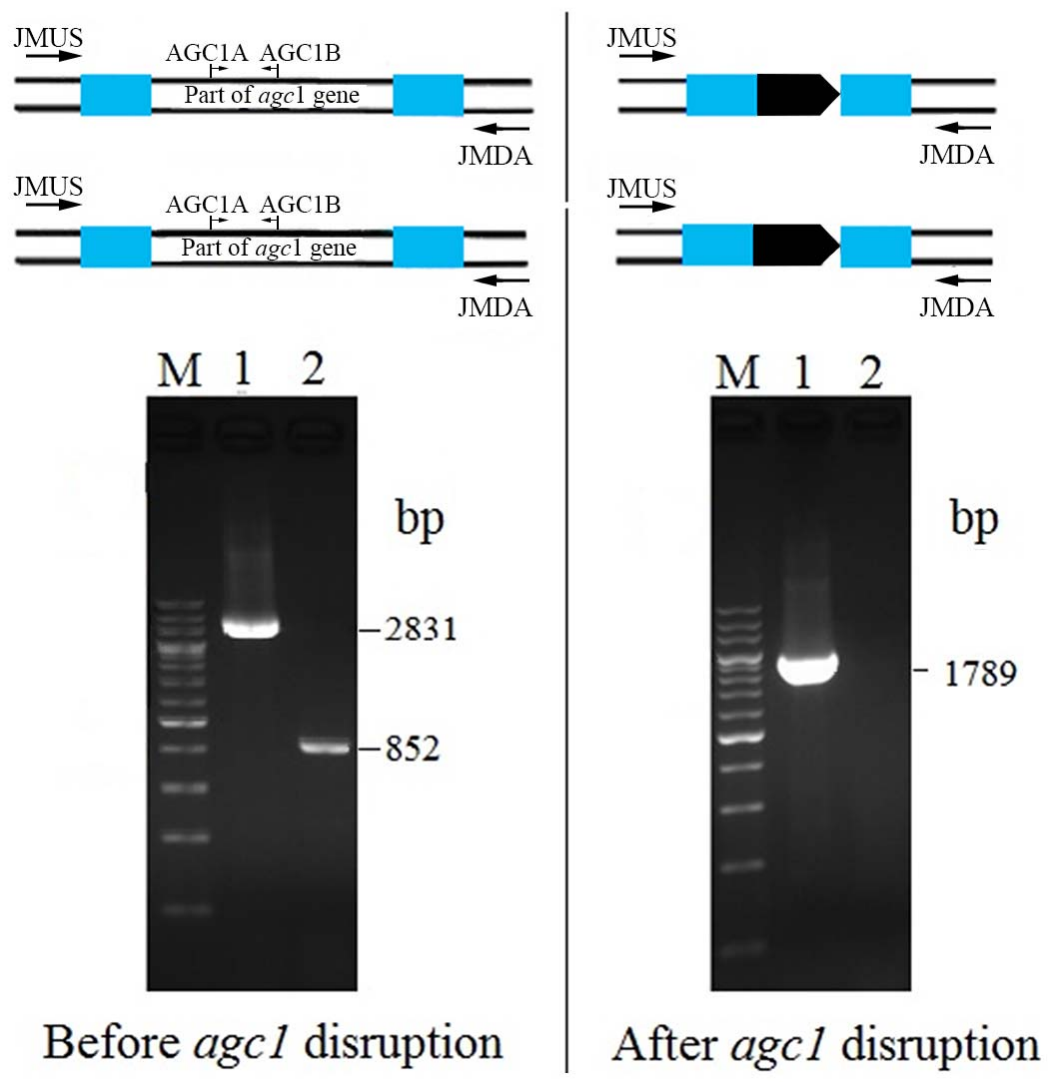
PCR detection of *agc1* gene in yeast BY4741 before and after the disruption procedure. The primer positions were labeled onto the schematic diagrams above the electropherograms. M, 200; 1, PCR product with the primer set JMUS and JMDA; 2, PCR product with AGC1A and AGC1B. PCR amplification with the first primer set yielded a 2831 bp band before while 1789 bp after *agc1* disruption. Meanwhile, PCR with the second primer set gave rise to an 852 bp band before but no product was detected after *agc1* disruption.

Two primer pairs were used to evaluate the knocking-out of *agc1* gene. The primers JMUS and JMDA were located in the upstream and downstream of the *agc1* gene sequence to be disrupted, while the primers AGC1A and AGC1B within the disrupted *agc1* sequences (Fig. 2, Table 1). Successful knocking-out of the *agc1* gene gave rise to a deduced 1789 bp product on PCR amplification with JMUS and JMDA while no product was observed when amplified with the primer set AGC1A and AGC1B.

### Plasmid Construction and Transformation

The normal citrin-coding sequence (NM_014251.2) was amplified with the primers PSLCF and PSLCR (Table 1), then the PCR product was recombined into expression plasmid pYX212 (Novagen) via *Eco*RI/*Hind*III, generating the vector of pYX212-CITRIN. The novel missense mutation was introduced into the wild type *SLC25A13* cDNA by overlap-extension PCR with the complementary primers V01F and V01R (Table 1) according to the literature [34]. Following mutagenesis, the generated variants were cloned into pYX212, constituting the vector pYX212-mutant. Then, transformation of BY*agc1*Δ strains with different plasmids including empty vector (pYX212), citrin control (pYX212-CITRIN) and the mutant plasmid (pYX212-mutant) were performed, respectively, by the improved lithium acetate method [33], and the positive clones were screened with the selection medium of SD-URA without uracil.

### Growth Ability and Statistics

The growth abilities were tested for the yeast strains of BY4741 (WT), the *agc1*-abrogated strain (BY*agc1*Δ), and BY*agc1*Δ strains transformed with the empty vector, citrin control and mutant plasmids, respectively, by using acetate as the unique carbon source. Growth tests were initiated with late log precultures grown on minimal medium (MM) [0.67% yeast nitrogen base with ammonium sulphate (YNB, Difco), 0.2% glucose as carbon source and 30 µg•ml^−1^ L-leucine, 20 µg•ml^−1^ L- methionine, 10 µg•ml^−1^ L-histidine, 20 µg•ml^−1^ uracil]. The precultures were diluted with synthetic acetate (SA) medium containing 100 mM sodium acetate, pH 5 [24], [35] until a final optical density of 5×10^−3^ at OD_600_ was reached. Growth tests were monitored by measuring the OD_600_ at an interval of 12 hours.

The data were analyzed by means of one-way ANOVA followed by the Bonferroni method to compare the differences in the mean values among the different groups above, with *P*<0.05 as the significance criteria.

## Results

### Clinical Findings

A male infant at the age of 4.5 months was referred to our hospital due to prolonged jaundice over 4 months. Mild jaundice appeared at the second day after birth and lasted without any alleviation by 1.5 months of age. Then the infant was admitted to the local hospital where physical examination revealed mildly jaundiced skin and sclera, and slightly enlarged liver 1.5 cm below the right costal margin. On biochemical analysis, elevated serum levels of alanine transaminase (ALT), gamma-glutamyl transpeptidase (GGT), total bilirubin (TBil), direct bilirubin (DBil), and total bile acid (TBA) were discovered (Table 2), indicating the existence of cholestatic liver disease. Urinary gas chromatography-mass spectrometry (GC-MS) analysis unveiled large quantity of 4-hydroxyphenyllactate (4HPL) and 4-hydroxyphenylpyruvate (4HPPV), while tandem mass spectrometry (MS-MS) analysis of serum amino acid revealed elevated levels of citrulline, threonine, arginine, tyrosine and methionine. No evidences of specific pathogens such as cytomegavirus, Epstein-Barr virus and tuberculosis were found. The diagnosis was idiopathic infantile hepatitis, and breastfeeding was stopped whist lactose-free formula introduced. He was discharged when the TBil was lowered to the level of 116.2 µmol/L at the age of 2 months. However, due to persistent jaundice in the later 2.5 months, the infant was referred to our hospital for further investigation. As the third child of a non-consanguineous couple, the patient was born at the gestational age of 38^+6^ weeks with the birth weight 2.6 kg. He had two healthy elder sisters and family history of any genetic disease was denied.

**Table 2:** Alterations of the serum biochemical indices in the patient.

Ages (Months)	1.5 M	2 M^a^	2.5 M	3 M	4 M	4.5 M^b^	5.5 M	10 M
ALT (5–40 U/L)	38	40	45	60	83	66	50	41
AST (5–40 U/L)	95	96	83	67	101	74	60	39
GGT (8–50 U/L)	242	233	320	222	217	176	238	23
ALP (20–500 U/L)	1480	1030	–	632	638	759	595	338
TP (60.0–83.0 g/L)	44.3	41.5	37.4	39.5	43.1	42.9	52.9	61.6
Alb (35.0–55.0 g/L)	30.5	27.5	27.7	29.0	33.3	31.9	37.4	46.1
Glb (20.0–30.0 g/L)	13.8	14.0	9.7	10.5	9.8	11.0	15.5	15.5
Tbil (2–19 µmol/L)	150.3	116.2	96.9	72.6	66.9	50.4	15.3	2.9
Dbil (0–6 µmol/L)	66.4	65.3	60.1	54.0	49.2	37.1	11.5	1.2
Ibil (2.56–20.9 µmol/L)	83.9	50.9	36.8	18.6	17.7	13.3	3.8	1.7
TBA (0–10 µmol/L)	354	227.8	230.4	153.8	66.4	39.9	40.6	2.6

*ALT* alanine transaminase; *AST* aspartate transaminase; *GGT* gamma-glutamyl transpeptidase; *ALP* alkaline phosphatase; *TP* total protein; *Alb* albumin; *Glb* globulin; *Tbil* total bilirubin; *Dbil* direct bilirubin; *Ibil* indirect bilirubin; *TBA* total bile acid.

a When breastfeeding was stopped, whist lactose-free formula introduced.

b When lactose-free and MCT-enriched therapeutic formula was introduced.

Physical examination in our hospital revealed the weight 5.9 kg (−2.0 SD), length 59 cm (−2.8 SD), and head circumference 40 cm (−1.8 SD). A chubby face and mildly-jaundiced skin and sclera were observed. The lungs were clear on auscultation, and no abnormal heart sound or murmur was heard. No abdominal distention, but a soft liver was palpable 3 cm below the costal margin on the right midclavicular line. Biochemical analysis still revealed elevated ALT, DBil, GGT and TBA (Table 2). Based on the history, physical findings and laboratory evidences, the infant was suspected to have NICCD, and *SLC25A13* gene analysis was then performed to confirm the diagnosis. A lactose-free and MCT-enriched therapeutic formula was introduced. As a result, his physical and biochemical conditions were improved gradually, and on following-up at his age of 10 months, the chubby face, jaundice and liver enlargement were resolved, with the weight 10.3 kg (+0.6 SD), length 71.0 cm (−1.4 SD) and head circumference 46.5 cm (+0.5 SD), indicating a successful catch-up growth. No serum biochemical abnormalities were observed (Table 2).

### *SLC25A13* Gene Mutations

**Figure 3:**
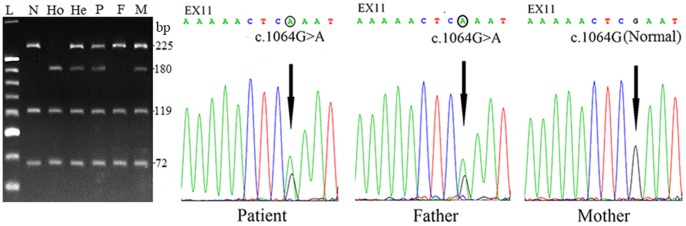
*SLC25A13* gene analysis in the family with a NICCD infant. The electrophoresis on the left illustrated the screening of the c.615+5G>A mutation by mean of a PCR-RFLP approach. The PCR products using the primer pair IVS5NF (5′-tgagggcttgttagatcaagat-3′) and IVS6NB (5′-ttacccagacaacaaattaacct-3′) were digested by TasI. L, 20 bp DNA ladder marker; N, Normal control; Ho, homozygous control; He, heterozygous control; P, Patient; F, Father of the patient; M, Mother of the patient. Note that the patient carried a maternally-inherited mutation c.615+5G>A. *SLC25A13* gene sequencing results on the right demonstrated that the patient habored a c.1064G>A mutation which was inherited from his father.

High-frequency mutation screening and direct sequencing revealed the patient a compound heterozygote of a maternally-inherited mutation c.615+5G>A and a novel missense mutation c.1064G>A (p.Arg355Gln), which was inherited from his father (Fig. 3). No carrier of this novel mutation was found in 50 control individuals, indicating a frequency less than 1%.

### Bioinformatic Analyses

**Figure 4:**
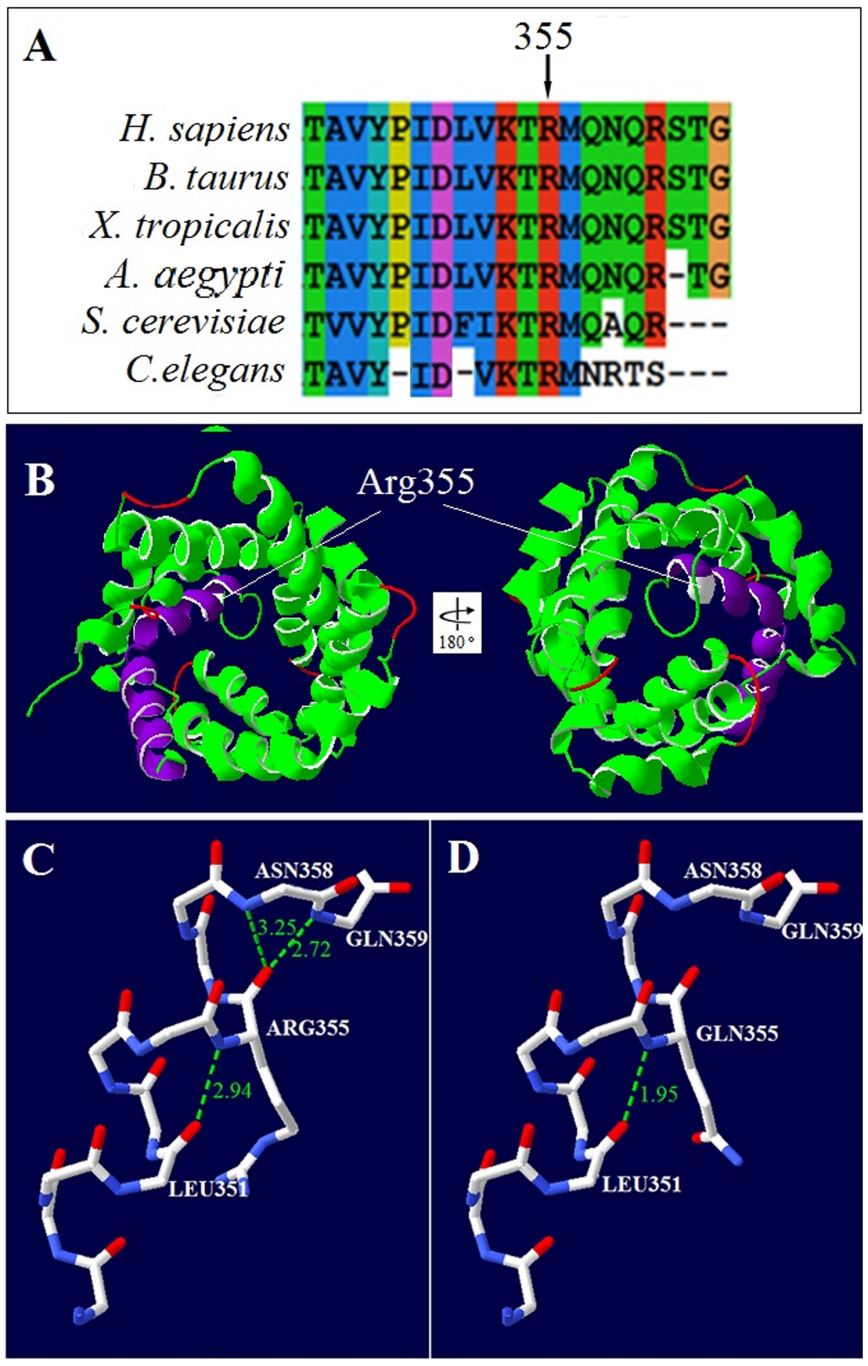
Comparative alignment of homologous proteins and structural alteration of citrin protein due to the novel mutation p.ARG355GLN. **A**. Comparative alignment of the homologous proteins in diverse species. The arrow showed the amino acid position affected by the novel mutation. Protein sequences were collected from *Human* (*H. sapiens*), *Cow* (*B. Taurus*), *Xenopus tropicalis* (*X. tropicalis*), *Aedes aegypti* (*A.aegypti*), *Yeast* (*S. cerevisiae*) and *Caenorhabditis elegans* (*C.elegans*), respectively. **B**. Ribbon model of the 6 transmembrane-spanning helices in citrin protein, which was constructed using bovine mitochondrial ADP/ATP carrier monomers (2c3eA) [28] as a template. The two views represented 180° rotation of the model. The ribbon in purple stood for the first transmembrane-spanning helix and the lines in red for the predicted β-turns. The residue in white represented the affected amino acid position. The ball-and-stick models in Figures **C** and **D** illustrated the backbone of the residues 348 to 359 with and without the mutation, with the short bars in white, red and blue standing for the carbon, oxygen and nitrogen atoms, respectively. Only the side-chain of residue 355 was shown. The dashed lines in green illustrated the potential hydrogen bonds from the residue 355, and the digits in green indicated the hydrogen bond distance (Å).

Comparative alignment of homologous proteins from yeast to human documented a conserved amino acid residue ARG355 in citrin protein (Fig. 4A). On structural model analysis, this residue was located within the first transmembrane-spanning helix of citrin protein (Fig. 4B), and the mutation p.Arg355Gln shortened the distance of the H-bond between residue 355 and LEU351 from 2.94 Å to 1.95 Å, and destructed the other two H-bonds between this residue and ASN358 and GLN359 (Fig. 4C–D). The hydrogen bond alterations predictively changed the spatial structure of the transmembrane-spanning helix, and thus affected the AGC function of citrin protein. Furthermore, functional prediction of the variant p.Arg355Gln by Polyphen-2 produced a value of 1.000, and the probability value is >0.999 on MutationTaster analysis, both suggesting a disease-causing mutation.

### Transcripts from the *SLC25A13* Allele Harboring the c.615+5G>A Mutation

**Figure 5:**
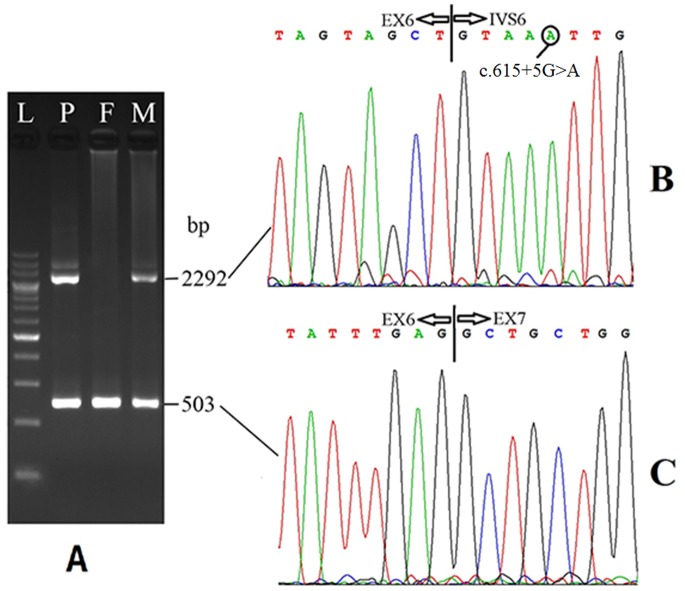
Sequencing results of the RT-PCR products. **A.** Electrophoresis of the PCR products with cDNA from PBLs as the templates. L, 200; P, Patient; F, Father of the patient; M, Mother of the patient. The 2292 bp and 503 bp products were sequenced, and the segmental sequencing results were illustrated in figures **B** and **C**, respectively. The 503 bp product had normal *SLC25A13* cDNA sequence, while the 2292 bp product retained the entire intron 6 due to the c.615+5G>A mutation.

On RT-PCR product electrophoresis, the patient and his mother both had two bands of 503 bp and 2292 bp, while his father just one band of 503 bp (Fig. 5A). The sequencing results demonstrated that the 503 bp product had expected normal sequence (Fig. 5C), while the entire intron 6 (1789 bp) of *SLC25A13* gene, along with the mutation c.615+5G>A, was retained in the 2292 bp product (Fig. 5B), strongly indicating that this variation was indeed a splice-site *SLC25A13* mutation. This concept was confirmed by further studies on 5 additional NICCD patients having the same mutation (results not shown). The aberrant mRNA molecule with intron 6 retention, *r*.[615+1_615+1789ins; 615+5g>a] (GenBank accession number: KJ128074), caused a frame shift from codon 206, added 6 amino acids, and produced a premature termination (TAA from the inserted sequence) at codon 212, thus forming a truncated citrin molecule p.Ala206Valfs212X.

### Knocking-out of the *agc1* Gene in Yeast Strain

Using the Cre-loxP recombination system, the *agc1* gene of *Saccharomyces cerevisiae* BY4741 was knocked out following two rounds of deletion procedures (Fig. 1). PCR detection of normal and *agc1*Δ strains were showed in Figure 2. The size of the PCR product amplified with the primers JMUS and JMDA were shortened from normal 2831 bp to 1789 bp after *agc1* gene disruption, and no PCR product from *agc1* gene was detected when amplified with the primers AGC1A and AGC1B. These results indicated that a fragment of *agc1* gene was deleted successfully.

### Functional Effect of the Novel Missense Mutation

**Figure 6:**
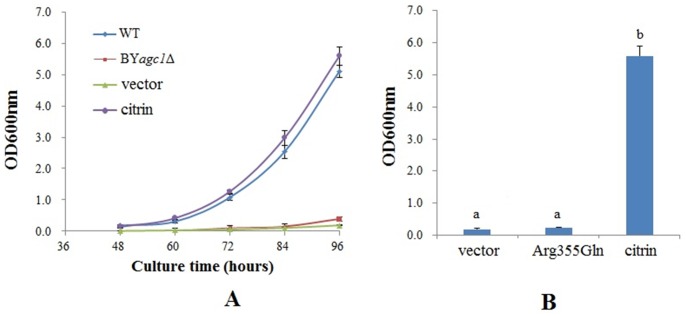
Growth properties of different yeast strains. **A,** wild type BY4741 (WT), strains with *agc1* gene disrupted (BY*agc1*Δ), BY*agc1*Δ strains transformed with empty plasmid pYX212 (vector) and normal control pYX212-CITRIN (citrin). The results were means ± SD of six repeated experiments. **B,** Growth abilities of the *agc1*Δ yeast strains. Strains of vector, citrin and Arg355Gln (BY*agc1*Δ strains transformed with mutant recombinant pYX212-mutant) was cultured in SA medium for 96 hours, and their growth abilities were monitored by measuring OD_600 nm_. The results were means ±SD of six repeated experiments, and different letters above the bars indicated statistically significant difference from each other (*P*<0.05).

The recombinant of pYX212 vector and the citrin variant with mutation c.1064G>A was named as pYX212-mutant. The growth abilities of the different yeast models were illustrated in Figure 6. BY*agc1*Δ cells had very poor growth ability with acetate as the unique carbon source, which could be restored after the transformation with the plasmid pYX212-CITRIN but not with the empty pYX212 vector (Fig. 6A). After growing for 96 hours, the cell density of the mutant group (Arg355Gln) did not demonstrate significant difference from that of the empty vector group, but both of them were significantly lower than that of the citrin control (Fig. 6B). These results indicated that the mutation p.Arg355Gln caused complete loss of the AGC function of citrin protein.

## Discussion

The growth retardation, prolonged jaundice, liver enlargement and the biochemical alterations indicating cholestatic liver disease were all non-pathognomonic in this infant. As mentioned above, however, citrin plays important roles as the liver-type AGC2 in a diversity of metabolic pathways such as aerobic glycolysis, gluconeogenesis and urea cycle, and thus it is not surprising that citrin deficiency could be reflected by the metabolic changes in NICCD. Actually, the metabolome findings in the infant in this paper, including the large excretion of 4HPL and 4-HPPV in the urine and the alterations of serum amino acids as well, provided valuable evidences strongly suggestive of NICCD [8]. *SLC25A13* genetic analysis (Fig. 3) unveiled a compound heterozygote of c.615+5G>A and c.1064G>A (p.Arg355Gln). The former was a common *SLC25A13* mutation, but the latter, so far as we know, has never been reported in any other references. Its frequency less than 1% indicated a novel mutation, and further bioinformatic analyses in this paper supported its pathogenicity by different tools including comparative alignment of homologous proteins, 3D structural analysis in silico and functional prediction using Polyphen-2 and MutationTaster.

Lactose-free and/or MCT-enriched therapeutic formulas have increasingly been reported to be effective on NICCD [7], [8], [36]–[38]. The clinical and biochemical improvement in this infant provided further evidences supporting this concept. Actually, galactitol and galactonate, the well-known metabolome makers for galactosemia, could be detected quite commonly in NICCD [8], indicating the derangement of galactose metabolism in such cases. Since galactitol was one of the substrates that caused the pathological abnormalities in classical galactosemia [39], [40], the effectiveness of lactose restriction might be attributed to the alleviation of the toxicity of galactitol on hepatocytes. On the other hand, MCT could be better absorbed and transported via the portal vein, and then more rapidly oxidized in comparison with long chain triglycerides (LCTs) [41]–[43]. Since the absorption of fat is bile acid-dependent, the well absorption of MCTs could thus reduce the burden of the liver to synthesize and excrete bile salt into the gut. Moreover, the increased cytosolic NADH/NAD^+^ ratio in hepatocytes were a key pathophysiologic alteration in citrin deficiency [44], [45]. This change could result in an energy shortage in the liver due to the impairment of glycolysis, and the therapeutic effect of MCT on NICCD might occur via a supply of acetyl-CoA, FADH2 and NADH to hepatic cells as energy sources [37].

Our study in this paper documented c.615+5G>A a real splice-site mutation that led to retention of the entire intron 6 in *SLC25A13* cDNA (Fig. 5). Pre-mRNA splicing involves the recognition of exon-intron junctions by the spliceosome and intron excision through a two-step transesterification reaction [46], [47], relying on conserved sequence elements at both ends of introns, termed splice sites [48], [49]. The most conserved 5′ Splice sites (5′ss) positions lies at the first two intronic nucleotides (GT or GU, +1 and +2), and the next most conserved 5′ss positions (>75% in humans) are −1G (the last exonic nucleotide) and +5G [48], [50]. This could be the reasonable explanation for intron 6 retention in cDNA due to c.615+5G>A in *SLC25A13* gene. The retention may lead to production of a premature termination codon that trigger nonsense-mediated mRNA decay (NMD), a surveillance mechanism that selectively degrades nonsense mRNAs [51]–[53], and thus the transcripts from the *SLC25A13* allele with c.615+5G>A were degraded soon after transcription, making its detection rather difficult in liver specimens or fibroblast cells. In this paper, the finding of the aberrant *SLC25A13* transcript *r*.[615+1_615+1789ins; 615+5g>a] (GenBank accession number: KJ128074) in PBLs of our NICCD infant might be partially attributed to the freshness of the blood samples for the RT-PCR process, and the difference of *SLC25A13* expression between PBLs and other tissues might be another likely contributing factor. Actually, the alternative splice variants (ASVs) with *r.*213_328del took account for over half among all *SLC25A13* transcripts in PBLs [16], constituting a transcriptional feature that had never been found in hepatocytes.

Missense mutations accounted for the largest proportion in the *SLC25A13* mutation list. Actually, there were 32 missense variations out of the total 84 possibly pathogenic *SLC25A13* variations identified in CD patients (Present study, [13], [22]). However, the pathogenicity of most missense mutations remains an unresolved issue due to the lack of functionally analytic evidences. Since human AGC2 was capable of functionally replacing the yeast gene *agc1* [35], a viable and simple model using yeast *agc1*Δ mutant for the functional analysis of human citrin variants was established very recently [24]. This model was utilized in our study to analyzed the effect of the novel c.1064G>A mutation on AGC2 function of citrin protein. As illustrated in Figure 6, c.1064G>A caused complete loss of AGC2 function of citrin protein, and therefore could be considered as a deleterious mutation. Besides the indirect bioinformatic evidences, this finding provided direct eukaryotic evidence for the pathogenicity evaluation of the missense mutation, and added further experience to the limited functional analysis of *SLC25A13* variation in NICCD patients.

In conclusion, a NICCD infant was definitely diagnosed as a compound heterozygote of c.615+5G>A and a novel c.1064G>A mutation by means of a clinical, genetic, transcriptional and functional investigation. Our findings provided direct laboratory evidences supporting the splice-site nature of the c.615+5G>A mutation, and the novel c.1064G>A variation, which proved a pathogenic mutation bioinformatically and functionally, expanded the *SLC25A13* mutation spectrum.

## References

[1] Kobayashi K, Sinasac DS, Iijima M, Boright AP, Begum L, et al (1999) The gene mutated in adult-onset type II citrullinaemia encodes a putative mitochondrial carrier protein. Nat Genet 22: 159–163. 10369257 10.1038/9667

[2] Kobayashi K, Saheki T (2003) Aspartate glutamate carrier (citrin) deficiency. In: Broer S, Wagner CS, editors. Membrane Transporter Diseases, NY: Kluwer Academic/Plenum Publishers, New York, 147–160.

[3] Palmieri L, Pardo B, Lasorsa FM, del Arco A, Kobayashi K, et al (2001) Citrin and aralar1 are Ca^2+^-stimulated aspartate/glutamate transporters in mitochondria. EMBO J 20: 5060–5069. 11566871 10.1093/emboj/20.18.5060 PMC125626

[4] Saheki T, Kobayashi K (2005) Physiological role of citrin, a liver-type mitochondrial aspartate-glutamate carrier, and pathophysiology of citrin deficiency. Recent Res Devel Life Sci 3: 59–73.

[5] Saheki T, Kobayashi K, Iijima M, Horiuchi M, Begum L, et al.. (2004) Adult-onset type II citrullinemia and idiopathic neonatal hepatitis caused by citrin deficiency: involvement of the aspartate glutamate carrier for urea synthesis and maintenance of the urea cycle. Mol Genet Metab (Suppl 1): S20–S26. 10.1016/j.ymgme.2004.01.006 15050970

[6] Saheki T, Kobayashi K, Iijima M, Li MX, Horiuchi M, et al.. (2006) Pathophysiology of citrin deficiency. In: Haussinger D, Kircheis G, Schliess F, editors. Hepatic Encephalopathy and Nitrogen Metabolism. Dordrecht, Netherlands: Springer, 320–328.

[7] Ohura T, Kobayashi K, Tazawa Y, Abkawa D, Sakamoto O, et al (2007) Clinical pictures of 75 patients with neonatal intrahepatic cholestasis caused by citrin deficiency. J Inherit Metab Dis 30: 139–144. 17323144 10.1007/s10545-007-0506-1

[8] Song YZ, Li BX, Chen FP, Liu SR, Sheng JS, et al (2009) Neonatal intrahepatic cholestasis caused by citrin deficiency: clinical and laboratory investigation of 13 subjects in mainland of China. Dig Liver Dis 41: 683–689. 19185551 10.1016/j.dld.2008.11.014

[9] Jiang GY, Cheng ZM, Liu KS (2012) Neonatal intrahepatic cholestasis caused by citrin deficiency: a histopathologic study of 10 cases. Zhonghua Bing Li Xue Za Zhi 41: 452–455. [Article in Chinese]. 10.3760/cma.j.issn.0529-5807.2012.07.005 22932455

[10] Okano Y, Kobayashi K, Ihara K, Ito T, Yoshino M, et al (2013) Fatigue and quality of life in citrin deficiency during adaptation and compensation stage. Mol Genet Metab 109: 9–13. 23453692 10.1016/j.ymgme.2013.01.020

[11] Yazaki M, Kinoshita M, Ogawa S, Fujimi S, Matsushima A, et al (2013) A 73-year-old patient with adult-onset type II citrullinemia successfully treated by sodium pyruvate and arginine. Clin Neurol Neurosurg 115: 1542–1545. 23369404 10.1016/j.clineuro.2012.12.027

[12] Song YZ, Deng M, Chen FP, Wen F, Guo L, et al (2011) Genotypic and phenotypic features of citrin deficiency: five-year experience in a Chinese pediatric center. Int J Mol Med 28: 33–40. 21424115 10.3892/ijmm.2011.653

[13] Song YZ, Zhang ZH, Lin WX, Zhao XJ, Deng M, et al (2013) SLC25A13 Gene Analysis in Citrin Deficiency: Sixteen Novel Mutations in East Asian Patients, and the Mutation Distribution in a Large Pediatric Cohort in China. PLoS One 8: e74544. 24069319 10.1371/journal.pone.0074544 PMC3777997

[14] Saheki T, Inoue K, Ono H, Tushima A, Katsura N, et al (2011) Metabolomic analysis reveals hepatic metabolite perturbations in citrin/mitochondrial glycerol-3-phosphate dehydrogenase double-knockout mice, a model of human citrin deficiency. Mol Genet Metab 104: 492–500. 21908222 10.1016/j.ymgme.2011.08.015

[15] Kobayashi K, Saheki T, Song YZ (2012). Citrin Deficiency, In: Pagon RA, Bird TD, Dolan CR, Stephens K, (Eds.), GeneReviews™ [Internet]. Seattle (WA): University of Washington, Seattle, 1993-. 2005 Sep 16 [Updated 2012 Jan 5].

[16] Zhang ZH, Lin WX, Deng M, Zhao XJ, Song YZ (2012) Molecular analysis of *SLC25A13* gene in human peripheral blood lymphocytes: Marked transcript diversity, and the feasibility of cDNA cloning as a diagnostic tool for citrin deficiency. Gene 511: 227–234. 23022256 10.1016/j.gene.2012.09.049

[17] Tabata A, Sheng JS, Ushikai M, Song YZ, Gao HZ, et al (2008) Identification of 13 novel mutations including a retrotransposal insertion in SLC25A13 gene and frequency of 30 mutations found in patients with citrin deficiency. J Hum Genet 53: 534–545. 18392553 10.1007/s10038-008-0282-2

[18] Dimmock D, Kobayashi K, Iijima M, Tabata A, Wong LJ, et al (2007) Citrin deficiency: a novel cause of failure to thrive that responds to a high-protein, low-carbohydrate diet. Pediatrics 119: e773–e777. 17332192 10.1542/peds.2006-1950

[19] Fu HY, Zhang SR, Wang XH, Saheki T, Kobayashi K, et al (2011) The mutation spectrum of the SLC25A13 gene in Chinese infants with intrahepatic cholestasis and aminoacidemia. J Gastroenterol 46: 510–518. 20927635 10.1007/s00535-010-0329-y

[20] Tokuhara D, Iijima M, Tamamori A, Ohura T, Takaya J, et al (2007) Novel diagnostic approach to citrin deficiency: analysis of citrin protein in lymphocytes. Mol Genet Metab 90: 30–36. 17092749 10.1016/j.ymgme.2006.09.009

[21] Lu YB, Kobayashi K, Ushikai M, Tabata A, Iijima M, et al (2005) Frequency and distribution in East Asia of 12 mutations identified in the SLC25A13 gene of Japanese patients with citrin deficiency. J Hum Genet 50: 338–346. 16059747 10.1007/s10038-005-0262-8

[22] Chen R, Wang XH, Fu HY, Zhang SR, Abudouxikuer K, et al (2013) Different regional distribution of SLC25A13 mutations in Chinese patients with neonatal intrahepatic cholestasis. World J Gastroenterol 19: 4545–4551. 23901231 10.3748/wjg.v19.i28.4545 PMC3725380

[23] Fiermonte G, Parisi G, Martinelli D, De Leonardis F, Torre G, et al (2011) A new Caucasian case of neonatal intrahepatic cholestasis caused by citrin deficiency (NICCD): a clinical, molecular, and functional study. Mol Genet Metab 104: 501–506. 21914561 10.1016/j.ymgme.2011.08.022

[24] Wongkittichote P, Tungpradabkul S, Wattanasirichaigoon D, Jensen LT (2013) Prediction of the functional effect of novel SLC25A13 variants using a *S. cerevisiae* model of AGC2 deficiency. J Inherit Metab Dis 36: 821–830. 23053473 10.1007/s10545-012-9543-5

[25] Lin WX, Zhang ZH, Deng M, Cai XR, Song YZ (2012) Multiple ovarian antral follicles in a preterm infant with neonatal intrahepatic cholestasis caused by citrin deficiency: a clinical, genetic and transcriptional analysis. Gene 505: 269–275. 22710133 10.1016/j.gene.2012.06.012

[26] Schwede T, Kopp J, Guex N, Peitsch MC (2003) SWISS-MODEL: An automated protein homology-modeling server. Nucleic Acids Res 31: 3381–3385. 12824332 10.1093/nar/gkg520 PMC168927

[27] Bordoli L, Kiefer F, Arnold K, Benkert P, Battey J, et al (2009) Protein structure homology modeling using SWISS-MODEL workspace. Nat Protoc 4: 1–13. 19131951 10.1038/nprot.2008.197

[28] Nury H, Dahout-Gonzalez C, Trézéguet V, Lauquin G, Brandolin G, et al (2005) Structural basis for lipid-mediated interactions between mitochondrial ADP/ATP carrier monomers. FEBS Lett 579: 6031–6036. 16226253 10.1016/j.febslet.2005.09.061

[29] Adzhubei IA, Schmidt S, Peshkin L, Ramensky VE, Gerasimova A, et al (2010) A method and server for predicting damaging missense mutations. Nat Methods 7(4): 248–249. 20354512 10.1038/nmeth0410-248 PMC2855889

[30] Schwarz JM, Rödelsperger C, Schuelke M, Seelow D (2010) MutationTaster evaluates disease-causing potential of sequence alterations. Nat Methods 7: 575–576. 20676075 10.1038/nmeth0810-575

[31] Güldener U, Heck S, Fielder T, Beinhauer J, Hegemann JH (1996) A new efficient gene disruption cassette for repeated use in budding yeast. Nucleic Acids Res 24: 2519–2524. 8692690 10.1093/nar/24.13.2519 PMC145975

[32] Gueldener U, Heinisch J, Koehler GJ, Voss D, Hegemann JH (2002) A second set of loxP marker cassettes for Cre-mediated multiple gene knockouts in budding yeast. Nucleic Acids Res 30: e23. 11884642 10.1093/nar/30.6.e23 PMC101367

[33] Gietz RD, Schiestl RH, Willems AR, Woods RA (1995) Studies on the transformation of intact yeast cells by the LiAc/SS-DNA/PEG procedure. Yeast 11: 355–360. 7785336 10.1002/yea.320110408

[34] Ho SN, Hunt HD, Horton RM, Pullen JK, Pease LR (1989) Site-directed mutagenesis by overlap extension using the polymerase chain reaction. Gene 77: 51–59. 2744487 10.1016/0378-1119(89)90358-2

[35] Cavero S, Vozza A, del Arco A, Palmieri L, Villa A, et al (2003) Identification and metabolic role of the mitochondrial aspartate-glutamate transporter in Saccharomyces cerevisiae. Mol Microbiol 50: 1257–1269. 14622413 10.1046/j.1365-2958.2003.03742.x

[36] Song YZ, Wen F, Chen FP, Kobayashi K, Saheki T (2010) Neonatal intrahepatic cholestasis caused by citrin deficiency: efficacy of therapeutic formulas and update of clinical outcomes. Jpn J Inherit Metab Dis 26: 57–69.

[37] Hayasaka K, Numakura C, Toyota K, Kimura T (2012) Treatment with lactose (galactose)-restricted and medium-chain triglyceride-supplemented formula for neonatal intrahepatic cholestasis caused by citrin deficiency. JIMD Rep 2: 37–44. 23430852 10.1007/8904_2011_42 PMC3509838

[38] Vitoria I, Dalmau J, Ribes C, Rausell D, García AM, et al (2013) Citrin deficiency in a Romanian child living in Spain highlights the worldwide distribution of this defect and illustrates the value of nutritional therapy. Mol Genet Metab 110: 181–183. 23835251 10.1016/j.ymgme.2013.06.011

[39] Bosch AM (2006) Classical galactosaemia revisited. J Inherit Metab Dis 29: 516–525. 16838075 10.1007/s10545-006-0382-0

[40] Ning C, Reynolds R, Chen J, Yager C, Berry GT, et al (2000) Galactose metabolism by the mouse with galactose-1-phosphate uridyltransferase deficiency. Pediatr Res 48: 211–217. 10926297 10.1203/00006450-200008000-00015

[41] Bach AC, Babayan VK (1982) Medium-chain triglycerides: an update. Am J Clin Nutr 36: 950–962. 6814231 10.1093/ajcn/36.5.950

[42] Lehner F, Demmelmair H, Röschinger W, Decsi T, Szász M, et al (2006) Metabolic effects of intravenous LCT or MCT/LCT lipid emulsions in preterm infants. J Lipid Res 47: 404–411. 16299352 10.1194/jlr.M500423-JLR200

[43] Roman C, Carriere F, Villeneuve P, Pina M, Millet V, et al (2007) Quantitative and qualitative study of gastric lipolysis in premature infants: do MCT-enriched infant formulas improve fat digestion? Pediatr Res 61: 83–88. 17211146 10.1203/01.pdr.0000250199.24107.fb

[44] Saheki T, Kobayashi K (2002) Mitochondrial aspartate glutamate carrier (citrin) deficiency as the cause of adult-onset type II citrullinemia (CTLN2) and idiopathic neonatal hepatitis (NICCD). J Hum Genet 47: 333–341. 12111366 10.1007/s100380200046

[45] Saheki T, Kobayashi K, Iijima M, Moriyama M, Yazaki M, et al (2005) Metabolic derangements in deficiency of citrin, a liver-type mitochondrial aspartate-glutamate carrier. Hepatol Res 33: 181–184. 16199199 10.1016/j.hepres.2005.09.031

[46] Hastings ML, Krainer AR (2001) Pre-mRNA splicing in the new millennium. Curr Opin Cell Biol 13: 302–309. 11343900 10.1016/s0955-0674(00)00212-x

[47] Brow DA (2002) Allosteric cascade of spliceosome activation. Annu Rev Genet 36: 333–360. 12429696 10.1146/annurev.genet.36.043002.091635

[48] Sheth N, Roca X, Hastings ML, Roeder T, Krainer AR, et al (2006) Comprehensive splice-site analysis using comparative genomics. Nucleic Acids Res 34: 3955–3967. 16914448 10.1093/nar/gkl556 PMC1557818

[49] Wahl MC, Will CL, Lührmann R (2009) The spliceosome: design principles of a dynamic RNP machine. Cell 136: 701–718. 19239890 10.1016/j.cell.2009.02.009

[50] Roca X, Krainer AR, Eperon IC (2013) Pick one, but be quick: 5′ splice sites and the problems of too many choices. Genes Dev 27: 129–144. 23348838 10.1101/gad.209759.112 PMC3566305

[51] Chang YF, Imam JS, Wilkinson MF (2007) The nonsense-mediated decay RNA surveillance pathway. Annu Rev Biochem 76: 51–74. 17352659 10.1146/annurev.biochem.76.050106.093909

[52] Green RE, Lewis BP, Hillman RT, Blanchette M, Lareau LF, et al.. (2003) Widespread predicted nonsense-mediated mRNA decay of alternatively-spliced transcripts of human normal and disease genes. Bioinformatics (Suppl 1): i118–121. 10.1093/bioinformatics/btg1015 12855447

[53] Lewis BP, Green RE, Brenner SE (2003) Evidence for the widespread coupling of alternative splicing and nonsense-mediated mRNA decay in humans. Proc Natl Acad Sci U S A 100: 189–192. 12502788 10.1073/pnas.0136770100 PMC140922

